# A Deep Learning Approach for Detecting Copy Number Variation in Next-Generation Sequencing Data

**DOI:** 10.1534/g3.119.400596

**Published:** 2019-08-27

**Authors:** Tom Hill, Robert L. Unckless

**Affiliations:** 4055 Haworth Hall, The Department of Molecular Biosciences, University of Kansas, 1200 Sunnyside Avenue, Lawrence, KS 66045.

**Keywords:** coverage, deletion, duplication, machine-learning, next-generation sequencing

## Abstract

Copy number variants (CNV) are associated with phenotypic variation in several species. However, properly detecting changes in copy numbers of sequences remains a difficult problem, especially in lower quality or lower coverage next-generation sequencing data. Here, inspired by recent applications of machine learning in genomics, we describe a method to detect duplications and deletions in short-read sequencing data. In low coverage data, machine learning appears to be more powerful in the detection of CNVs than the gold-standard methods of coverage estimation alone, and of equal power in high coverage data. We also demonstrate how replicating training sets allows a more precise detection of CNVs, even identifying novel CNVs in two genomes previously surveyed thoroughly for CNVs using long read data.

Copy number variation (CNV) of DNA sequences is responsible for functional phenotypic variation in many organisms, particularly when it comes to causing or fighting diseases (Sturtevant 1937; Inoue and Lupski 2002; Rastogi and Liberles 2005; Freeman *et al.* 2006; Redon *et al.* 2006; Schrider *et al.* 2013; Unckless *et al.* 2016). Additionally, recent studies have found that duplications and deletions are an important type of mutations with functional consequences for evolution to act upon (Korbel *et al.* 2007; Schrider *et al.* 2013; Zichner *et al.* 2013; Rogers *et al.* 2014; Cardoso-Moreira *et al.* 2016). Despite its importance, properly detecting copy number variants is difficult and so the extent that CNVs contribute to phenotypic variation has yet to be fully ascertained (Redon *et al.* 2006; Zichner *et al.* 2013; Chakraborty *et al.* 2018). This detection difficulty is due to challenges in aligning CNVs, with similar copies of duplications being combined in assembling Sanger-sequencing genomes (Redon *et al.* 2006; Chakraborty *et al.* 2018). Additionally, mapping short-read NGS data to a reference genome lacking the duplication will combine the two gene copies as a single copy (Redon *et al.* 2006; Ye *et al.* 2009; Abyzov *et al.* 2011; Rausch *et al.* 2012; Layer *et al.* 2014). Several tools have been developed to detect these CNVs in next-generation sequencing (NGS) data, but for proper accuracy, they require high coverages of samples (for the detection of split-mapped reads, or better estimations of relative coverage), long-reads (able to span CNVs) or computationally intensive methods (Redon *et al.* 2006; Ye *et al.* 2009; Abyzov *et al.* 2011; Rausch *et al.* 2012; Layer *et al.* 2014; Chen *et al.* 2016; Chakraborty *et al.* 2018). This limits the ability to detect CNVs between samples sequenced to relatively low coverages, with short reads, or on lower quality genomes.

The recent development of numerous machine learning techniques in several aspects of genomics suggests a role for machine learning in the detection of copy number variants (Larrañaga *et al.* 2006; Libbrecht and Noble 2015; Sheehan and Song 2016; Schrider *et al.* 2018; Schrider and Kern 2018). Contemporary machine learning methods can classify windows across the genome associated with selective sweeps with surprising accuracy, even using lower quality data (Larrañaga *et al.* 2006; Libbrecht and Noble 2015; Kern and Schrider 2018). Additionally, supervised machine learning techniques are generally less computationally intensive than other unsupervised machine learning methods such as Approximate Bayesian Computation, because the user provides a training set for the supervised detection of classes (Beaumont *et al.* 2002; Jensen *et al.* 2008; Beaumont 2010; Bouckaert *et al.* 2014; Schrider and Kern 2018).

Here we introduce a novel deep-learning based method for detecting copy number variation (primarily duplications and deletions), named ‘**du**plication and **de**letion Classifier using **M**achine **L**earning’ (dudeML). We outline our rationale for the statistics used to detect CNVs and the method employed, in which we calculate relative coverage changes across a genomic window (divided into sub-windows) which allows for the classification of window copy number using different machine learning classifiers. Using both simulated and known copy number variants, we show how dudeML can correctly detect copy number variants, outperforming basic coverage estimates alone and other CNV predictors when read depth is low.

## Methods and Model

### Machine learning method

**Figure 1:**
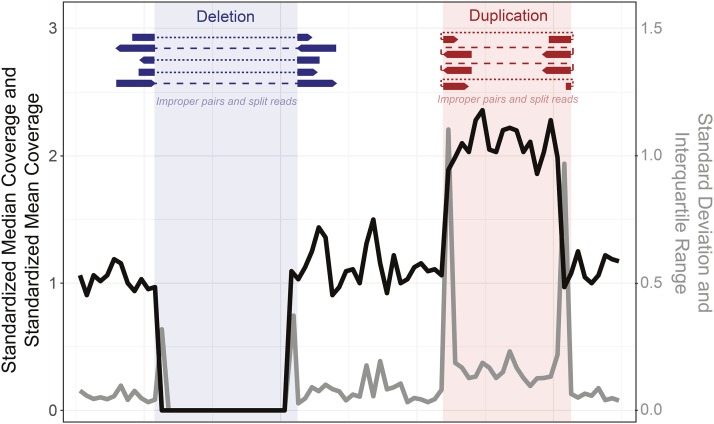
Schematic demonstrating the rationale behind each statistic used to initially determine the presence/absence of each copy number variant. We expect the standardized median and mean coverage (black line) to increase in duplications (red) and decrease in deletions (blue). We expect the standard deviation and interquartile range of the standardized coverage to greatly increase at the edges of CNVs (gray line). At the borders of CNVs we also expect an increase in reads with supplementary alignments and improperly mapped read pairs, specifically across the edges of deletions (dark blue) or within a tandemly duplicated region (dark red).

Inspired by recent progress in machine learning for population genomics (Larrañaga *et al.* 2006; Pedregosa *et al.* 2011; Libbrecht and Noble 2015; Schrider and Kern 2016; Sheehan and Song 2016; Kern and Schrider 2018; Schrider and Kern 2018), we sought to develop a method to accurately and quickly classify the presence or absence of copy number variants in genomic windows using a supervised machine learning classifier. Based on previous software and methods for copy number detection (Ye *et al.* 2009; Abyzov *et al.* 2011; Rausch *et al.* 2012; Chen *et al.* 2016), we identified a number of values that may help determine if a duplication or deletion is present in a particular genomic window. We reasoned that both standardized median and mean coverage should indicate if a window is an outlier from the average coverage of a scaffold (Figure 1, black), and that the interquartile range and standard deviation in standardized coverage of a window will increase in regions with higher coverage, decrease in regions with lower coverage and increase dramatically at copy number variant (CNV) edges due to rapid shifts in coverage (Figure 1, gray). Here we define standardization as dividing the coverage by the mean or median of the coverage of all bases on the contig, so the standardized coverage is distributed around 1, with a minimum of 0 and no limit to the maximum. Another component of some CNV detection algorithms are unidirectional split mapped reads and the mapping of improper pairs surrounding or within a CNV which also indicate the breakpoint of a structural variant such as a deletion or tandem duplication (expected at the red/blue borders in Figure 1) (Ye *et al.* 2009; Palmieri *et al.* 2014).

We used these measures across a set of sub-windows within a window to define the copy number and CNV class of the focal sub-window at the center, using a classifier (Figure 1). Initially, we sought to identify which of the measures (and in what windows) are most useful for determining the presence or absence of a copy number variant, relative to a reference genome. To do this, we simulated tandem duplications and deletions (100-5000bp) across the *Drosophila melanogaster* reference chromosome 2L. We then simulated 100bp paired-end reads for this chromosome using WGsim (Li 2011) (parameters: -e 0.05 -d 500 -1 100 -2 100 -r 0.01 -R 0.15) and mapped these to the standard reference 2L using BWA and SAMtools (Li and Durbin 2009; Li *et al.* 2009) in two forms, either ignoring repetitive windows based on calls from RepeatMasker (Smit and Hubley 2013-2015) (parameters: -s -gff -gccalc -lib [custom] -no_is -e ncbi -norrna -pa 5) or without masking and including all windows. We also simulated a second set of CNVs and related short read data as a test set. We considered samples mapped to the repeat masked genome and excluding masked windows to be ‘no repeats’, while samples which included the repetitive regions were labeled ‘with repeats’.

To identify candidate CNVs, we calculated the values described above (standardized mean and median, standard deviation and interquartile range of the standardized coverage, and number of improper pairs) in sub-windows multiple times, each time for a different sub-window size (ranging from 10bp to 1000bp, sliding the same distance). We reformatted the data to vectors including the statistics for a focal sub-window and 10 sub-windows upstream and downstream of the focal sub-window, creating a set of statistics describing the 20 sub-windows around a focal sub-window, for every window in the chromosome. We then assigned each window a class, based on the known copy number and known class (deletion-containing, duplication-containing or normal) for the focal sub-window. We trained a random forest classifier with 100 estimators (Liaw and Wiener 2002; Pedregosa *et al.* 2011) to extract the features necessary to classify the presence or absence of a CNV in the central sub-window. We also repeated this process to identify important statistics as coverage of the training set decreases. The scripts and a tutorial for this process are available at https://github.com/tomh1lll/dudeml, including the tool for detecting CNVs.

### Classifier optimization

We sought to determine how window size (10 - 1000bp), number of windows (1 - 41), mean coverage of data (0.2 - 40) the number of individuals in a sequenced pool (1 - 40), and how the machine learning algorithm (Random Forest 100 estimators and 500 estimators, Extra Trees 100 and 500 estimators, Decision Tree, and Convolutional Neural Network classifiers) (Pedregosa *et al.* 2011) affects the ability to correctly classify a CNV in simulated data. In all cases using the same 2-D vector of values as input (Pedregosa *et al.* 2011). In each case we changed only one variable, otherwise coverage was set at 20-fold, window-size was set at 50bp, the number of sub-windows each side was set to 5 and the algorithm was set as Random Forest (100 estimators). For all comparisons (coverages, window sizes, number of windows or algorithm comparisons) we counted the number of True and False positive CNVs and estimated a receiver operating characteristic curve (Brown and Davis 2006).

We used bedtools (Quinlan and Hall 2010) and RepeatMasker (Smit and Hubley 2013-2015) to identify regions on chromosome 2L without high levels of repetitive content. We then simulated 2000 duplications and 2000 deletions across these regions, varying in size between 100bp and 5000bp. To assess a machine learning classifier’s ability to detect CNVs across pooled data, we created a further subset of CNVs present at different frequencies in pools of chromosomes. We employed pools of 2 (the equivalent of sequencing an outbred diploid individual), 5, 10, 15, 20, 30 and 40 chromosomes, allowing the CNV to vary in frequency between 2.5% and 100% across samples, based on the number of chromosomes simulated (*e.g.*, a 50% minimum in a pool of 2 chromosomes, equivalent to a heterozygous CNV, and a 5% minimum in a pool of 20, equivalent to a singleton CNV in a pool of 20 chromosomes). This process was repeated twice to create independent test and training sets, both with known CNVs.

We generated chromosomes containing simulated CNVs and simulated reads for these chromosomes using WGsim (Li 2011) (parameters: -e 0.05 -d 500 -1 100 -2 100 -r 0.01 -R 0.15). We simulated reads to multiple median depths of coverage per base, between 0.2 to 40. We then combined all reads for each pool set and mapped these reads to the *D. melanogaster* Iso-1 reference 2L (dos Santos *et al.* 2015) using BWA and SAMtools (Li and Durbin 2009; Li *et al.* 2009).

For each data set, of varying window sizes, coverages and pool sizes, we then reformatted each window as described above to give the statistics for the focal window and 10 windows up and downstream, unless otherwise stated. For each training set, we gave each vector a class label, noting the presence of a duplication, deletion or neither. We also gave each vector a second-class label of copy number relative to the chromosome average: 0 for a fixed deletion, 0.5 for a deletion found in 50% of chromosomes, 1.75 for a duplication found in 75% chromosomes, 3 for a fixed duplication with 3 copies etc. We then used SKlearn to train a classifier based on the vectors assigned to each class (Pedregosa *et al.* 2011). The classifiers were then used to assign classes to windows in the test sets, which were then compared to their known designations to identify the true positive detection rate of each set.

Finally, we compared our classifier to several other CNV callers: Delly (Rausch *et al.* 2012), Pindel (Ye *et al.* 2009), iCopyDAV (Dharanipragada *et al.* 2018), CNVnator (Abyzov *et al.* 2011), using parameters best fitting for each given data set) as well as pure coverage for samples of decreasing coverage or increasing poolsize. We were unable to get iCopyDAV to function for samples with coverage less than an average of onefold and so did not include samples below this coverage.

### Examining the copy number estimation of dudeML *vs.* coverage-based estimates

We sought to compare the estimation of copy number variation of sequences using dudeML. For this we downloaded the genome of the large DNA virus Drosophila innubila Nudivirus (DiNV) from NCBI genome database: NC040699.1 (Hill and Unckless 2018) and created test and training sets for multiple copy numbers of DiNV (ranging from 0 to 50), relative to the coverage of 2L in *Drosophila melanogaster*. We then estimated copy number for each version of DiNV using dudeML and based on median coverage of DiNV divided by the median coverage of 2L, we also predicted copy number by fitting a loess regression on coverage *vs.* copy number in R (Team 2013). For these methods we then compared the rate of correct copy number estimation as copy number increases, and the mean difference in predicted and actual copy number as copy number increases.

### Testing the classifier on real data with known CNVs

To test the classifier using known variants from long-read sequencing data, we downloaded the *D. melanogaster* Iso-1 and A4 reference genomes (dos Santos *et al.* 2015; Chakraborty *et al.* 2018). Then, based on (Chakraborty *et al.* 2018), we extracted windows with known duplications and deletions relative to each other, for example a tandem duplication present in one genome but not the other would appear as a deletion of one copy of the sequence when reads for the non-duplicated strain are mapped to the genome containing the duplication. Mapping the corresponding short-read data for the duplicated strain to the non-duplicated strain genome would increase the coverage of the duplicated region. We downloaded short reads for each *D. melanogaster* genome (Iso-1: SRA ERR701706-11, A4: http://wfitch.bio.uci.edu/∼dspr/Data/index.html, ADL6/b3852 within SRA051316.tar) and mapped them to both the Iso-1 and A4 genomes separately using BWA MEM and SAMtools (Li and Durbin 2009; Li *et al.* 2009). Using the previously described methods, we calculated the statistics for each sub-window of each genome using bedtools and custom python scripts contained within dudeML. Using the training set described previously, we then classified each window of Iso-1 and A4 mapped to alternative reference and compared called CNVs to the previously detected CNVs.

For each dataset, we also simulated 100 independent training sets, which we used to test the effectiveness of bootstrapping the random forest classifier. Each window was reclassified for each bootstrap training set, which are then used to calculate the consensus state for each window and the proportion of bootstrap replicates supporting that states.

Finally, to validate any apparent ‘false-positive’ CNVs identified with our machine learning classifier, we downloaded pacific bioscience long read data for both Iso-1 and A4 (A4 PacBio SRA: SRR7874295 - SRR7874304, Iso-1 PacBio SRA: SRR1204085 - SRR1204696), and mapped this data to the opposite reference genome. For each high confidence (greater than 95% of bootstraps) ‘false-positive’ CNV, we manually visualized the PacBio data in the integrative genomics viewer (Robinson *et al.* 2011), looking for changes in coverage and split-mapped reads. For a randomly chosen group of these CNVs, we designed primers and confirmed CNVs using PCR (Data S1 & S2). We designed primer pairs around each CNV to assess product size differences between strains, as well as inside the CNV for strain specific amplification for deletions or laddering in the case of duplications. PCR products from primer sets in both Iso-1 and A4 were then run on a 2% gel using gel electrophoresis (Figure S9).

### Data availability

All data used in this manuscript are freely available and published online. dudeML and the simulated data used in this manuscript is available at https://github.com/tomh1lll/dudeml. The DiNV genome can be found on the NCBI genome repository under the accession GCA_004132165.1. The latest releases of both the Iso-1 and A4 *Drosophila melanogaster* genomes can be found on the NCBI genome database at: https://www.ncbi.nlm.nih.gov/genome/genomes/47. Short reads for each *D. melanogaster* genome can be accessed either through the NCBI SRA or on the DSPR online repository - Iso-1: SRA ERR701706-11, A4: http://wfitch.bio.uci.edu/∼dspr/Data/index.html, ADL6/b3852 within SRA051316.tar. Finally, long read data for both Iso-1 and A4 can be found on the NCBI SRA: A4 PacBio SRA: SRR7874295 - SRR7874304, Iso-1 PacBio SRA: SRR1204085 - SRR1204696. Supplemental material available at Figshare: https://doi.org/10.25387/g3.9735890.

## Results and Discussion

### A machine learning classifier can detect CNVs with high accuracy

**Figure 2:**
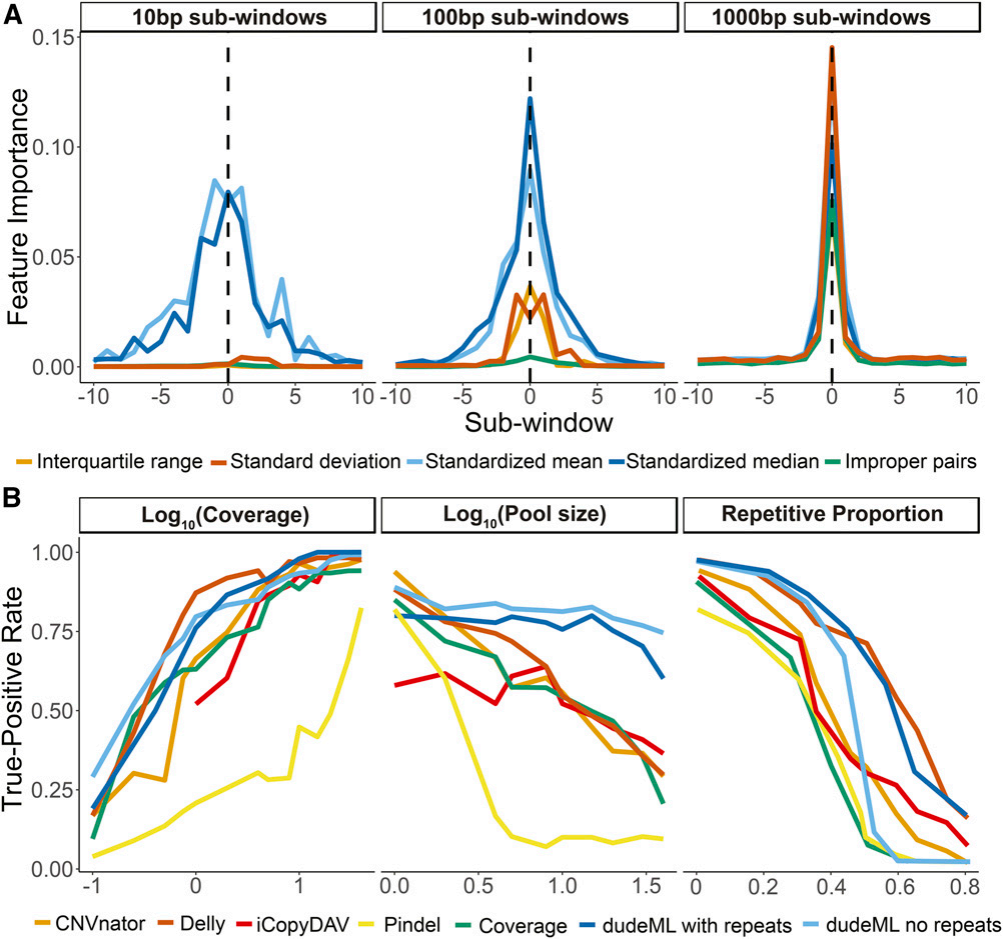
A. Relative contribution of each statistic to the classification of copy number variants, across windows in increasing distance from the focal sub-window (dashed lined), separated by sub-window size. B. Comparison of detection of copy number variants between other CNV detectors, pure coverage estimations and using dudeML for varying parameters. Detection rate decreases across all methods with decreasing coverage, with increasing pool sizes and increasing repetitive content of the focal region. Note that iCopyDAV requires a certain level of coverage to function correctly, so was not used below onefold coverage.

We sought to develop a rapid, simple and accurate classifier of copy number variants in next-generation sequencing data (Pedregosa *et al.* 2011; Schrider and Kern 2016; Schrider *et al.* 2018). First, we assessed how useful multiple statistics are in the detection of non-reference duplications and deletions in short-read next-generation sequencing data (Figure 1). We simulated short read data for a chromosome containing multiple insertions and deletions relative to a reference genome and mapped these reads to the original reference chromosome. We divided each genomic window into a set of sub-windows, centered on a focal sub-window of interest. For each sub-window we calculated standardized median and mean coverage, the standard deviation and interquartile range of the standardized coverage within each sub-window, and the standardized number of reads with supplementary alignments (*e.g.*, split mapped) and reads in improper pairs (with too large or small insert sizes or mapped to different chromosomes) across the sub-window. We reasoned that each of these statistics can signal the increase or decrease of copy number of a sequence relative to a reference genome (Figure 1, see Materials and Methods). We created a vector of these statistics for the focal sub-window and each sub-window surrounding it, giving a set of values summarizing the window. These vectors of statistics for windows with known copy number variants (CNVs) are then fed into a machine learning classifier, which identifies the values most important to the correct classification of the presence of a CNV and the correct estimation of copy number. For simplicity we will refer to this classifier as the **Du**plication and **De**letion Classifier using **M**achine **L**earning (dudeML) moving forward. The tool developed as a wrapper for the pipeline, instructions for installation, specifics of the pipeline for detecting copy number variants, and links to test data used in this manuscript are available at https://github.com/tomh1lll/dudeml. Using dudeML on high coverage (>20-fold), simulated copy number variants, we find that standardized median coverage and interquartile range, and standardized mean coverage and standard deviation across windows (per individual, across all sites in a sub-window) are important for classifying the focal sub-window (Figure 2A). Surprisingly, the number of reads with supplementary alignments (reads where two ends map to different regions of the genome) and improperly paired reads are relatively unimportant for finding CNVs for smaller sub-window sizes (Figure 2A, below 1000bp), but increases in importance as sub-window size increases (Figure 2A). Though the breadth of a distribution will vary depending on the window-size and mean size of the CNV, the most important sub-windows for classifying a CNV appear to be the focal sub-window and up to 5 windows up and downstream of the focal sub-window (Figure 2A). We also find different statistics have different contributions across different window sizes and different coverages. For example, larger windows are more likely to include the edges of the CNV so standard deviation and improper read pairs are more important for CNV classification in larger windows (Figure 2A). However, larger windows appear to have lower true-positive rates, again due to the increased chance of overlapping with repeat content (Figure S1, true-positive rate ∼ window size: GLM t-value = -2.968, p-value = 0.00303), which can be accommodated to some degree by including repeat content (Figure S4). Similarly, as coverage increases, the classifier relies less on standard deviation and standardized mean of the sub-windows surrounding the focal sub-window, and more on median, improper pairs and interquartile range of the focal sub-window (Figure S2 & S3).

We also compared different supervisor machine learning classifiers and found no significant differences between most classifiers (Figure S4), though the most successful classifier on simulated data were a Random Forest Classifier (true-positive rate ∼ classifier GLM t-value = 0.758, p-value = 0.765), with no difference in results between 100 and 500 estimators (Pedregosa *et al.* 2011). We incorporated all classifiers into dudeML as they may be more successful in conditions not tested here.

### CNV machine learning classifiers are relatively agnostic to coverage and can detect CNVs in pooled data with high accuracy

For simulated data of varying coverages containing known CNVs, we compared dudeML to the prediction of a CNV based on copy number alone (rounding the median coverage of the sub-window to the nearest whole value, divided by the average coverage of the chromosome), or other CNV detection software: Delly (Rausch *et al.* 2012), CNVnator (Abyzov *et al.* 2011), iCopyDAV (Dharanipragada *et al.* 2018) and Pindel (Ye *et al.* 2009). All methods appear to have decreasing rates of CNV detection as coverage decreases (Figure 2B, true-positive rate ∼ coverage: GLM t-value = 3.090, p-value = 0.0029), with dudeML having a high rate of success (Figure 2B), decreasing at a similar rate to Delly and CNVnator (Figure 2B, true-positive rate ∼ coverage * dudeML: GLM t-value = 1.001, p-value = 0.329), but having a high rate of success at drastically low coverage (0.1x). As most CNV detection programs rely on split-mapped reads of certain mapping orientations to detect copy number variants, low coverage data likely lacks a sufficient abundance of these reads for the correct detection of CNVs (Ye *et al.* 2009; Abyzov *et al.* 2011; Rausch *et al.* 2012). Similarly, the spurious nature of data at low coverages prevents pure relative coverage comparisons from being useful. With machine learning however, if the training data are like the sampled data, the classifier relies on thousands of similar examples in each state to more reliably predict the presence or absence of a CNV. In fact, correctly predicting a CNV in data of decreasing coverage with a poorly optimized training set has a similar success rate as pure-coverage alone (Figure S3), highlighting the importance of a training set as similar to the true data as possible.

We also investigated the detection of CNVs in genomic regions of increasing repetitive content using each method (using data at 20-fold coverage). CNVs found in highly repetitive regions are less likely to be correctly detected across all software formats (Figure 2B, true-positive rate ∼repetitive portion of window: GLM t-value = -0.4657, p-value = 1.54e-05), though dudeML factoring in repetitive content appears to have relatively (though not significantly) higher success than most other methods (true-positive rate ∼repetitive portion of window * dudeML: GLM t-value = 0.1328, p-value = 0.18639).

Often, populations are sequenced as pools of individuals instead of individually prepared samples because the approach reduces cost while still providing relatively high power for population genetic inference (Schlötterer *et al.* 2014). To asses dudeML’s ability to detect the correct number of copies of a gene in a population, we simulated CNVs at varying frequencies throughout pools of chromosomes. We generated simulated pools as both test data and training sets of 1 (haploid or inbred), 2 (diploid, 50% coverage), 5, 10, 20 and 40 chromosomes (pools at onefold coverage for each chromosome), again, we compared this to each other tools ability to detect the CNV and relative coverage estimates. As expected, as pool size increased the true positive rate decreased in all methods (Figure 2, true-positive rate ∼pool size: GLM t-value = -4.883, p-value = 1.20e-05). All other forms of detection decreased in detection rate at a much faster rate than dudeML in both forms (true-positive rate ∼pool size * dudeML: GLM t-value > 2.414, p-value < 0.019660).

### CNV machine learning classifiers can be optimized to drastically improve the quality of CNV detection, and should consider repetitive content and sample coverage

We next tested the extent that changing different parameters affected dudeML’s ability to correctly detect CNVs. We examined the effects of decreasing coverage, increasing sub-window size, increasing pool size and increasing the number of sub-windows on correctly classifying CNVs with dudeML, when including repetitive regions or ignoring them (Figures S4 & S5). As dudeML classifies windows as containing a CNV or not, while other classifiers call CNVs, we found both the true-positive rate for windows and for correct CNV calls.

As sub-window-size increases, the false-negative rate increases in the repeat masked sample, due to more windows overlapping with repetitive regions, excluding these windows from the analysis. In the repetitive sample, the false-positive rate increases as sub-window-size decreases due to more spurious coverage in the focal window affecting the detection of CNVs. Similar effects are seen when increasing the number of sub-windows in the repeat-masked sample, as smaller sub-window numbers with stochastic coverage can lead to more false-positives, while larger sub-window numbers have more windows ignored by the classifier due to overlaps with repeat content (Figures S4 & S5). We also tested classifiers of different coverages and sub-window sizes against samples with an incorrect/poorly optimized training set. We find that as training sets diverge in similarity from the actual data, the true-positive rate decreases dramatically (Figure S3, true-positive rate ∼ absolute(log10(classifier coverage) – log10(sample coverage)): GLM t-value = -14.147, p-value = 7.36e-32), giving the classifier a success rate like pure-coverage alone (Figure 2, Figure S1), highlighting the importance of a training set as like the true data as possible.

### dudeML also accurately predicts copy number of a gene target region compared to pure coverage, for copy number estimation of genes or viruses in a sample

We sought to examine further applications of dudeML. Alongside predicting if a window contains a CNV or not, dudeML also predicts the copy number of a window. We examined how well dudeML predicts copy numbers compared to copy number estimation from coverage alone (window coverage/median coverage) and compared to the predictions from a loess regression. As copy number increases, the success rate of all methods decreases (Figure S6, true copy number proportion ∼ copy number: GLM t-value = -10.639 p-value = 4.87e-12). At all copy numbers, dudeML has much higher true-positive rate and accuracy in predicting copy number (Figure S6, true copy number proportion ∼ copy number + dudeML: GLM t-value = 6.146 p-value = 7.12e-07), with success rate and accuracy decreasing much more slowly as copy number increases (Figure S6, true copy number proportion ∼ copy number * dudeML: GLM t-value = 2.616, p-value = 0.0134). In fact, even when incorrect, the copy number estimate of dudeML is much closer to the actual copy number than other methods (Figure S6). The success of dudeML in predicting copy number suggests it could be used to predict the copy number of genes with high variation in copy number, viral genome copies relative to host genome copy number, or copy number of RNA viruses relative to a housekeeping gene. Additionally, though better tools are available (Rahman *et al.* 2015; Nelson *et al.* 2017) to detect transposable element copy numbers, dudeML could be used for this purpose. dudeML could even be used for B chromosome copy number estimation, though may have issues depending on the window-size and the size of the usually poorly assembled B-linked contigs.

### Resampling increases CNV machine learning classifier accuracy

To further tune the accuracy of our classifier, we tested its effectiveness on the detection of copy number variants in real data, as opposed to simulated copy number variants in simulated reads (though with a classifier still using simulated CNVs and simulated data for training). We therefore downloaded two *Drosophila melanogaster* reference genomes – both assembled with long-read data – with identified duplications and deletions relative to each other (A4 and Iso-1) (Chakraborty *et al.* 2018). When data from one reference is mapped to the other, regions with copy number variants show signatures of changes in standardized coverage and standard deviation as seen in simulated data (Figure 1, Figure S7, Data S1). These real datasets slightly differ in their important features from the simulated data, in that the standardized mean and median hold slightly less importance, while standard deviation has slightly more importance in CNV prediction (Figure S7). However, this is not significantly different from the training sets generated with simulations (feature importance ∼ feature * real: GLM t-value = 0.331, p-value = 0.652).

As before we trained the classifier based on mean and median coverage, interquartile range and standard deviation of coverage and the number of improper pairs, for windows containing simulated CNVs and standard regions. We performed this analysis with and without repetitive regions in the simulated training set and the real data. We then predicted windows with duplications or deletions using a random forest approach (Pedregosa *et al.* 2011).

Strangely, and unseen in simulated examples, the proportion of false-positives was extremely high, with orders of magnitudes more false-positives compared to true-positives (Table 1). This number of false-positives is reduced by filtering out CNVs with low predicted probabilities (less than 95% probability), but still produced a similar number of false-positives to true-positives (Table 1, Data S3).

**Table 1:** The number of predicted copy number variants in each strain (relative to the alternate strain), compared to previously identified copy-number variants (Chakraborty *et al.* 2018), across differing numbers of bootstraps and probability cutoffs, including the true-positive rate (TPR) for each category. The total count of previously identified CNVs is also included in the table. Table contains CNVs called in repeat masked samples with repeat masked training set, therefore CNVs overlapping with TEs have been removed, all other permutations of CNV calls, with and without repeats and with differing numbers of bootstraps and filtering, can be found in Data S3

Number of classifiers (% bootstrap/probability cutoff)		Iso-1		A4	
	Predictions	Duplication	Deletion	Duplication	Deletion
1 (0)	True-positive	144	174	151	132
1 (0)	False-Positive	2489	3004	5462	2239
1 (0)	False-Negative	7	2	14	8
	**TPR**	**0.0537**	**0.0547**	**0.0268**	**0.0555**
1 (95)	True-positive	144	174	151	132
1 (95)	False-Positive	56	83	99	79
1 (95)	False-Negative	7	2	14	8
	**TPR**	**0.7024**	**0.7280**	**0.5720**	**0.6027**
10 (0)	True-positive	150	170	161	135
10 (0)	False-Positive	1516	2344	3064	1717
10 (0)	False-Negative	3	6	4	5
	**TPR**	**0.100**	**0.060**	**0.073**	**0.050**
10 (90)	True-positive	148	170	161	135
10 (90)	False-Positive	56	83	57	34
10 (90)	False-Negative	5	6	4	5
	**TPR**	**0.827**	**0.854**	**0.797**	**0.776**
100 (0)	True-positive	150	176	161	139
100 (0)	False-Positive	135	82	178	82
100 (0)	False-Negative	3	0	4	1
	**TPR**	**0.521**	**0.682**	**0.469**	**0.626**
100 (95)	True-positive	145	176	153	133
100 (95)	False-Positive	43	32	56	27
100 (95)	False-Negative	8	0	12	7
	**TPR**	**0.873**	**0.936**	**0.692**	**0.796**
	**Total**	**153**	**176**	**165**	**140**

**Figure 3:**
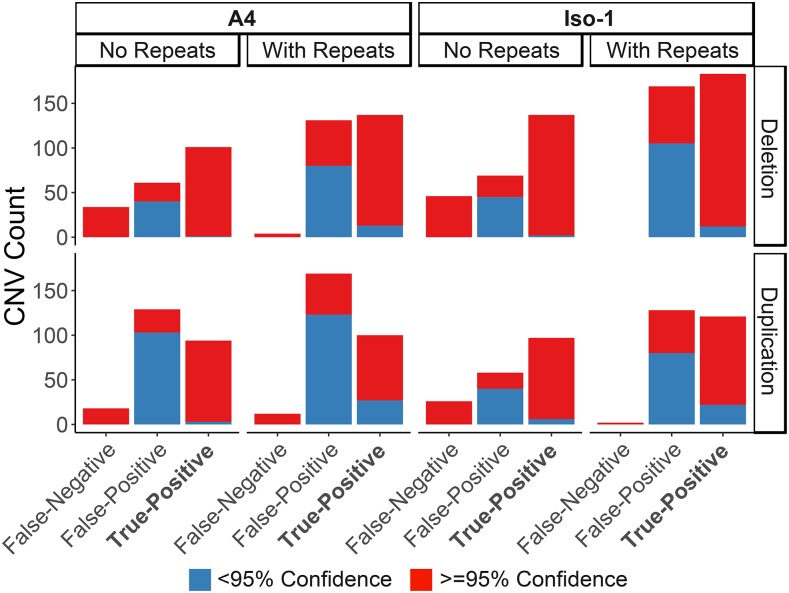
Number of CNVs detected in *Drosophila melanogaster* strains with known CNVs relative to each other after 100 bootstraps, for both samples including and excluding repeats. Note that the sample without repeats also include CNVs within masked regions (which we did not survey) as false-negatives. CNVs are grouped by their previously known detection in these strains (‘True-Positive’), their lack of knowledge in these strains (‘False-Positive’) and if known CNVs were missed (‘False-Negative’). CNVs are labeled based on the confidence in the calls, the proportion of bootstraps confirming them.

We suspected that artifacts and false CNVs may be caused by real structural variants and areas with inconsistent mapping rates that went undetected in the original simulated training set, consistent with this, twice as many false-positive CNVs are called when repetitive regions are included in both the samples and training sets (true-positive rate ∼ TE: GLM t-value = -0.279, p-value = 0.781, Figure 3, Data S3). We attempted to control for this by resampling across multiple training sets with independently generated CNVs. We generated 100 independent training sets across both the Iso-1 and A4 reference genomes to create 100 independent classifiers. Following this we performed a bootstrapping-like approach, predicting the copy number of each window 100 times using the 100 independent classifiers and taking the consensus of these calls. As the number of replicates increased, the false-positive rate drops dramatically with little effect on the true-positive rate (FPR ∼ replicates: GLM t-value = -5.309, p-value = 4.40e-06, Table 1, Figure 3, Data S3). As the probability value for CNV increases, the false-positive rate also decreases (FPR ∼ replicates: GLM t-value = -8.506, p-value = 1.65e-10). This did however remove some real duplications, so provides a conservative set of CNVs (Figure 3) (Chakraborty *et al.* 2018). This suggests that multiple independent training sets can remove any artifacts found in a single training set which may lead to false calls (Table 1, Figure 3).

Interestingly, including repetitive regions in the training set increases the detection rate of high confidence false-positives, and decreases the confidence in true-positive CNV calls (Figure 3), suggesting that even if repetitive regions are screened using dudeML, repetitive regions included in the training set results in decreased confidence in CNV calls (Figure S8).

As so many false-positives are found with high confidence across both samples, we next visually inspected the regions of the genome called as false-positive CNVs in at least 95 of 100 bootstraps (Figure S8): 99 duplications (23% of duplications called) and 59 deletions (15% of deletions called) across both strains). We extracted long reads (> 250bp) from PacBio data for both strains and mapped these to the opposite strain’s genome, which we then visualized in the integrative genomics viewer (Robinson *et al.* 2011). All false-positive CNVs examined show similar signatures to true-positive copy numbers (*e.g.*, split-mapped reads across regions of 0 coverage for deletions, and supplementary alignments of reads in regions of high coverage for duplications), suggesting that they are in fact real CNVs and not false-positives (18 examples given in Data S1). We further PCR validated 12 of these CNVs, chosen at random (Figure S8 & S9, Data S2). While we could validate all deletions, we found no length variation in PCR product for putative duplications for primers designed outside the duplication, which suggests if these duplications exist they may not be tandem duplications (which would produce a longer or laddered PCR product) and instead are *trans* duplications. Logically this would fit with the absence of these CNVs in the previous survey which searched for tandem duplications specifically (Chakraborty *et al.* 2018), while dudeML identifies duplications primarily based on coverage-based statistics and so is agnostic to *cis vs. trans* duplications. However, the lack of PCR verification could support these duplications as segregating within the originally sequenced line or are false-positives.

Based on these results, bootstrapping (and filtering low probability calls) appears to average over random effects of simulated training sets to improve the accuracy of CNV calls, removing a majority of false-positive CNVs called while not majorly affecting the true-positives (in fact improving their calls in some cases), allowing a more conservative assessment of the copy number variants found throughout an assessed strain. Most high confidence false-positives also appear to be actual CNVs, suggesting that dudeML can detect CNVs other tools miss – even using long read data.

### Conclusion

In summary, we have shown that machine learning classifiers, such as dudeML, perform quite well at detecting copy number variants in comparison to other methods, particularly in samples with reduced coverage or in pools, using statistics easily derived from the sample. These tools are not computationally intensive and can be used across many datasets to detect duplications and deletions for numerous purposes. We expect machine learning to provide powerful tools for bioinformatic use in the future.
